# Age‐Related Trends in Breast Cancer Incidence

**DOI:** 10.1155/ijbc/6629139

**Published:** 2026-04-24

**Authors:** Batool Mutar Mahdi, Noor Kamil Abbas, Haneen Khalid Hameed, Shahad Jamal Abdualdayeam

**Affiliations:** ^1^ HLA Research Unit, Department of Microbiology, Al-Kindy College of Medicine, University of Baghdad, Baghdad, Iraq, uobaghdad.edu.iq; ^2^ Department of Microbiology, Al-Kindy College of Medicine, University of Baghdad, Baghdad, Iraq, uobaghdad.edu.iq

**Keywords:** age, breast, tumor

## Abstract

**Background:**

Breast cancer is one of the diseases in which abnormal, mutated breast cells grow out of control and form tumors. If left unchecked, the tumors can spread throughout the body and become fatal.

**Aim of the Study:**

This study is aimed at assessing the combined effect of age‐specific trends over time on breast cancer.

**Materials and Methods:**

The cross‐sectional study included 100 patients diagnosed with breast cancer over a period extending from January 2024 to January 2025 at Al‐Amal Hospital in Baghdad. The data were collected from the records of the hospital, including the demographic ones, and another section of the data included the medical ones.

**Results:**

A total of 100 breast cancer patients were included in this study, with ages ranging from 25 to 75 years (mean ± SD : 50.47 ± 10.96 years). The highest percentage of breast cancer patients (39%) belonged to the 50–59 years age group, followed by 40–49 years (25%), while the lowest percentages were observed in the 21–29 years (5%) and 70–79 years (5%) categories. Menstrual history illustrated that 52% of patients were postmenopausal, while 44% were premenopausal, and 4% had irregular cycles. Most cancer patients were married (78%), while 11% were widowed, 8% were single, and 3% were divorced.

**Conclusions:**

This study highlights the demographic and clinical characteristics of breast cancer patients, emphasizing the predominance of cases in postmenopausal women and those residing in urban areas. It highlights the prevalence of advanced‐stage and metastatic breast cancer, emphasizing the urgent need for enhanced screening and early detection.

## 1. Introduction

Breast cancer is the most common tumor affecting women worldwide and caused 670,000 deaths globally in 2022 [1]. In developed countries like the United States and the United Kingdom, breast cancer is the second lethal cause of cancer in women; similarly, in low‐ and middle‐income countries, the mortality rate was also high due to the lack of resources for preventative screening and early detection of tumors and treatment [2]. Breast cancers occur in women with no specific risk factors other than sex and age; however, trends in breast cancer mortality and incidence vary substantially with age among different countries [3]. In some countries, the mortality decreased for women aged 55–69 years, while in other countries, the mortality rate decreased in women aged below 50 years [4]. Many countries demonstrated that the mortality rate increased in women older than 65 years in spite of screening methods and advanced treatment [5]. Analysis of breast cancer in the United States showed that mammogram screening decreased the mortality rate to 58%, treatment at Stages I and II decreased the mortality to 47%, and treatment for metastatic breast cancer was associated with a 29% decrease in the mortality rate [6]. These differences among countries in age‐related mortality trends may be related to differences in mammographic screening policies, ultrasound examination, and age‐related differences in uptake of drugs like tamoxifen, use of hormone replacement therapy, and use of oral contraceptive pills [7, 8]. Another test is the Tru‐Cut needle biopsy, which is superior to fine‐needle aspiration cytology in the detection of breast cancer in palpable breast masses with a low cost and low complication rate [9]. Increased breast density is associated with a possible increase in risk of tumor using different methods like ultrasound and mammogram, which play a role in estimating breast density [10]. In Iraq, breast cancer among young Iraqi women below 40 years had a high incidence rate of mortality but might be less aggressive than what is reported in western countries despite a high recurrence rate [11]. Another factor is obesity, which is associated with a decreased risk of breast cancer before menopause but an increased risk after menopause [12]. Accurate plans for breast cancer screening are important for planning future public health policy and resource distribution. In particular, accurate age‐specific plans to decrease mortality from breast cancer are essential for assessing cancer control programs like mammographic screening and improvements in treatment options.

This study assesses the combined effect of age‐specific trends over time on breast cancer mortality rate and estimating future trends of age‐specific decreased breast cancer mortality.

## 2. Materials and Methods

The cross‐sectional study population included 100 patients diagnosed with breast cancer over a period extending from January 2024 to January 2025 at Al‐Amal Hospital in Baghdad, Iraq. The minimum sample size was estimated using Cochran′s formula for prevalence studies, assuming a 50% expected proportion, 5% margin of error, and 95% confidence interval. The calculated minimum sample size was 96 participants, indicating that the inclusion of 100 patients in the present study provides sufficient statistical precision for the intended analyses. In this study, data extraction and entry were independently performed and cross‐checked by two investigators to minimize measurement error and ensure accuracy. The study was approved by the Scientific and Ethical Committee of Al‐Kindy Medical College and Medical City Hospital—Ministry of Health, under Approval No. 42598, dated 2‐12‐2024. Inclusion criteria encompassed all patients diagnosed with tumors of the breast at that period who consulted this hospital, while exclusion criteria applied to those with other types of tumors. The data were collected from the records of the hospital, including demographic ones that comprised age, address, marital status, period, blood group, family history, other diseases, and other sections of the data that included medical ones like stage of cancer, metastasis, and treatment modality such as surgery, chemotherapy, radiotherapy, immunotherapy, and recurrence. The data were stratified according to age and other variables.

### 2.1. Statistical Analysis

Data were collected and conducted using Microsoft Excel (2016) and SPSS (Version 28). Descriptive statistics were used to summarize demographic and clinical characteristics. Continuous variables were presented as mean ± standard deviation (SD) and compared between groups using the Student *t*‐test. Categorical variables were expressed as frequencies and percentages and analyzed using the chi‐square test or Fisher′s exact test when cell counts were small. The level of statistical significance was set at *p* < 0.05 for all tests.

## 3. Results

**Figure 1:**
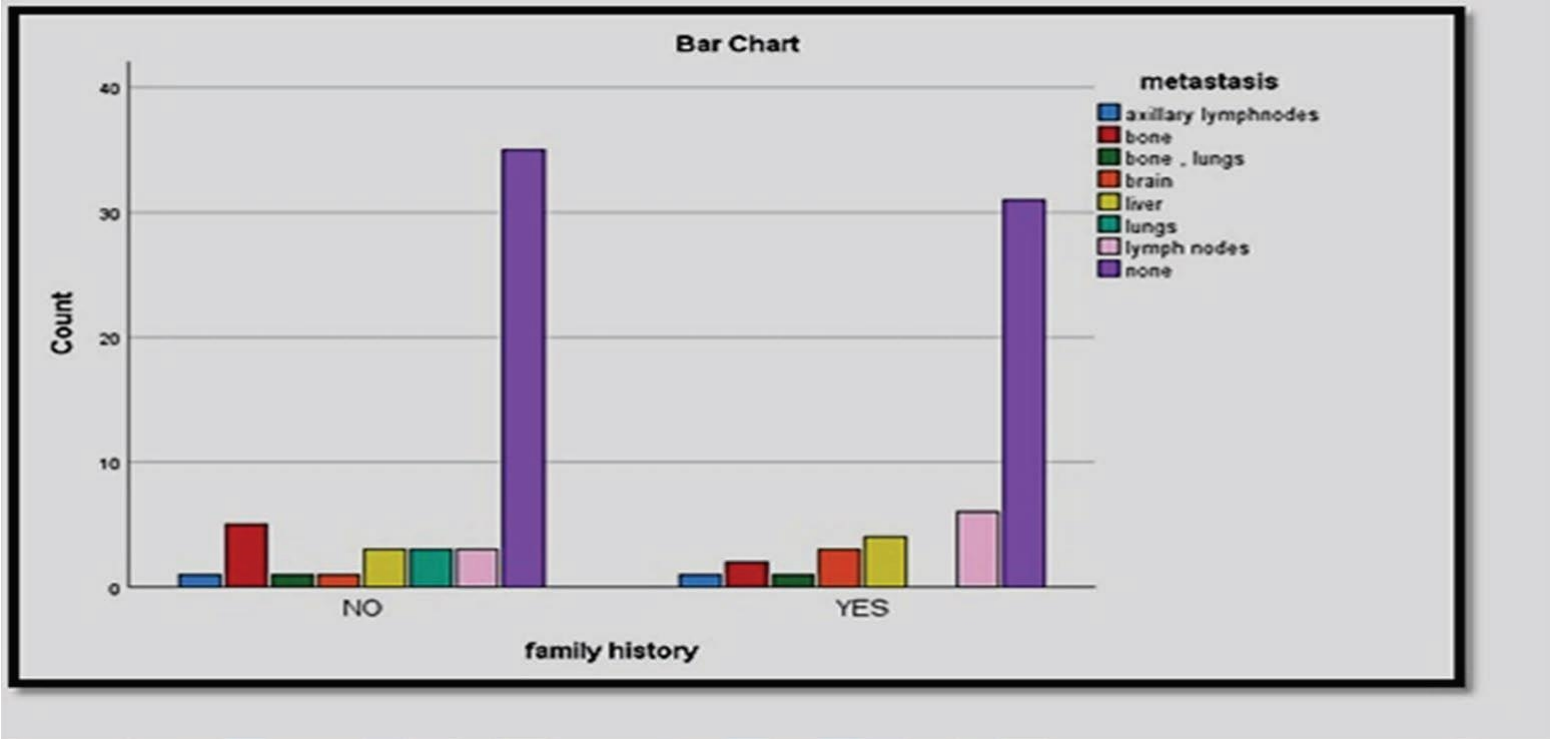
Metastasis of breast cancer patients.

**Figure 2:**
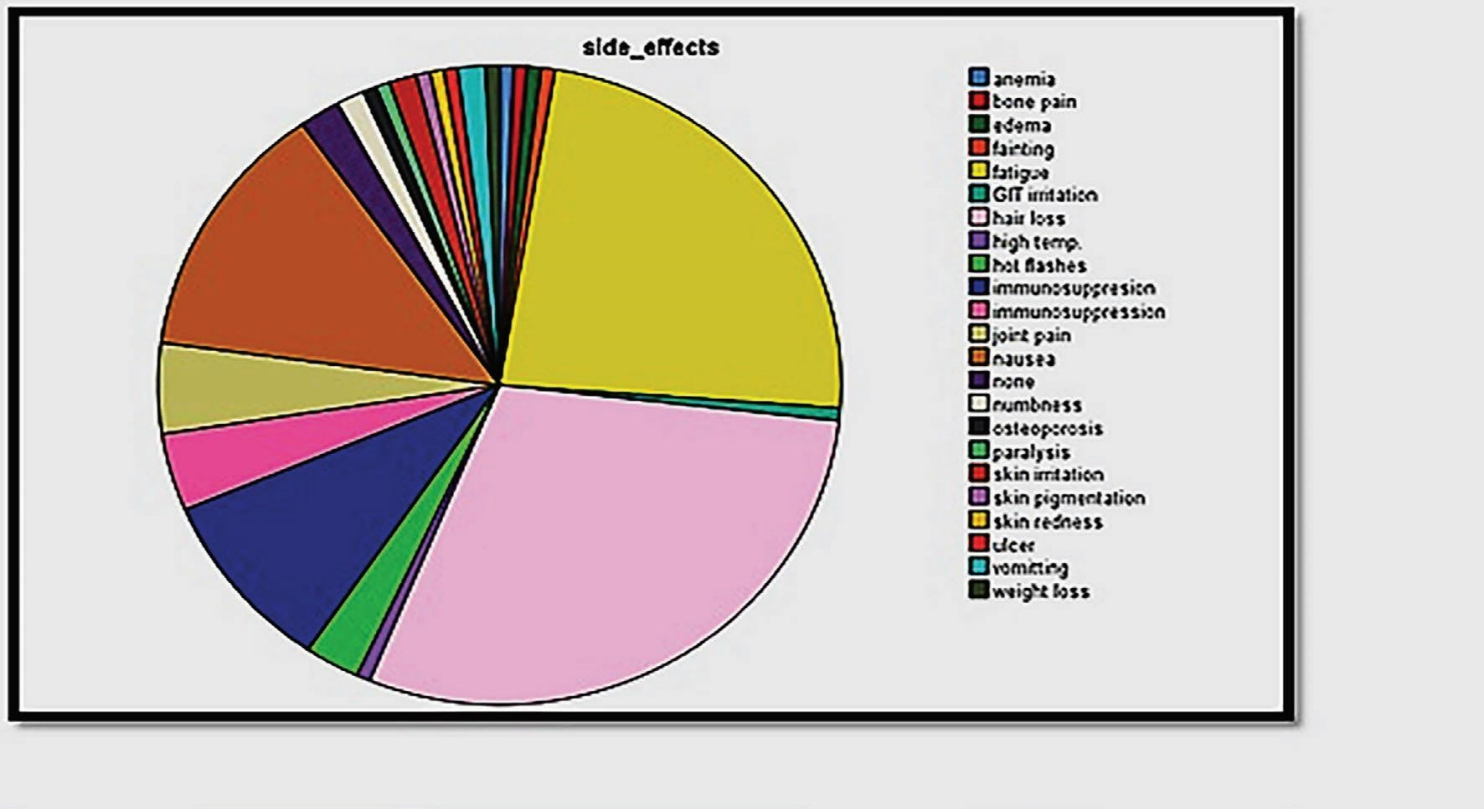
Side effects of the drug in patients with breast cancer.

A total of 100 breast cancer patients were included in this study, with ages ranging from 25 to 75 years (mean ± SD : 50.47 ± 10.96 years). The highest percentage of breast cancer patients (39%) belonged to the 50–59 years age group, followed by 40–49 years (25%), while the lowest percentages were observed in the 21–29 years (5%) and 70–79 years (5%) categories. Menstrual history illustrated that 52% of patients were postmenopausal, while 44% were premenopausal, and 4% had irregular cycles. Most cancer patients were married (78%), while 11% were widowed, 8% were single, and 3% were divorced. Regarding family planning, 44% of the patients reported not using contraceptives, while 29% used oral contraceptives, and smaller proportions used intrauterine devices (IUDs), condoms, or tubal ligation. Among the study population, 50% had no significant medical history, while hypertension (19%), diabetes (9%), and diabetes with hypertension (5%) were the most common comorbidities. Obesity, hypothyroidism, osteoporosis, and anemia were also reported but in lower frequencies. A family history of breast cancer was positive in 48% of patients, while 52% had no family history. Most patients (93%) were from Baghdad, while 7% were from other provinces. The significant *p* value (0.000) suggests a strong association between urban residency and breast cancer incidence (Table 1). The study classified breast cancer patients based on cancer stage, with the majority (48%) diagnosed at Stage II, followed by Stage I (21%), Stage III (19%), and Stage IV (12%). The patients had metastatic breast cancer to regional areas or distant spread mostly to axillary lymph nodes (Figure 1). The blood group distribution among breast cancer patients was O+ (17%) and A+ (15%) as the most common, whereas AB− (3%), B− (2%), and A− (2%) were the least common. Moreover, a significant proportion (37%) had an unknown blood group. All patients underwent computerized tomography (CT) and mammography, reflecting the standard diagnostic approach for breast cancer detection and staging. Mammography remains the gold standard for early detection, while CT scans assist in staging and evaluating metastasis. The most common treatment strategies included chemotherapy (50%) which is the primary systemic treatment for advanced/metastatic cases, surgery (37.1%) performed in operable cases for tumor removal, and radiotherapy (12.4%) for metastatic control, and the last mode of treatment was chemo‐biological (immunotherapy) (0.5%), which is an emerging therapy for specific subtypes. A 21% recurrence rate was observed, while 79% of patients remained recurrence‐free (Table 2). The most common side effects of the drugs were fatigue (36, 23.5%) and hair loss (45, 29.4%) (Figure 2).

**Table 1:** Demographic data of patients with breast cancer.

Variables		No.	%	*p*
Age (years)				
Mean ± SD	50.47 ± 10.96			
Range	25–75			
Age stratification	21–29	5	5	*p* ≤ 0.001
	30–39	12	12	
	40–49	25	25	
	50–59	39	39	
	60–69	14	14	
	70–79	5	5	
Menstrual history	Irregular cycle	4	4	*p* ≤ 0.001
	Postmenopausal	52	52	
	Premenopausal	44	44	
Marital status	Divorced	3	3.0	*p* ≤ 0.001
	Married	78	78.0	
	Single	8	8.0	
	Widowed	11	11.0	
Family planning	Contraceptive injection	3	3.0	*p* ≤ 0.001
	Intrauterine device (IUD)	9	9.0	
	IUD, tubal ligation	1	1.0	
	No contraceptive	44	44.0	
	Oral contraceptives	29	29.0	
	Oral contraceptives, IUD	4	4.0	
	Condom	5	5.0	
	Tubal ligation	5	5	
Medical history	Anemia	2	2.0	*p* ≤ 0.001
	Asthma, hypertension	1	1.0	
	Diabetes	9	9.0	
	Diabetes, hypertension	5	5.0	
	Diabetes, obesity	2	2.0	
	Hypertension	19	19.0	
	Hypothyroidism	2	2.0	
	Hysterectomy, diabetes	1	1.0	
	Insulin resistance	1	1.0	
	Polycystic ovary	3	3.0	
	Obesity	4	4.0	
	Osteoporosis	1	1.0	
	Negative medical history	50	50	
Family history	Yes	48	48	0.57
	No	52	52	
Address	Baghdad	93	93	*p* ≤ 0.001
	Other provinces	7	7	

Abbreviation: NS, nonsignificant.

**Table 2:** Characteristics of breast cancer.

Variables		No.	%	*p*
Stage of cancer	Stage I	21	21.0	1.04
	Stage II	48	48.0	
	Stage III	19	19.0	
	Stage IV	12	12	
Metastasis	Yes	100	100	Not applicable
	No	0	0	
Blood group	A−	2	2.0	*p* ≤ 0.001
	A+	15	15.0	
	AB−	3	3.0	
	AB+	6	6.0	
	B−	2	2.0	
	B+	12	12.0	
	O−	6	6.0	
	O+	17	17.0	
	Unknown	37	37.0	
Screening tests	Computerized tomography, mammography	100	00	
Treatment	Surgery	69	69	*p* ≤ 0.001
	Chemotherapy	93	93	
	Radiotherapy	23	23	
	Chemo‐biological (immunotherapy)	1	1	
Recurrence	No	79	79.0	*p* ≤ 0.001
	Yes	21	21.0	

## 4. Discussions

Breast cancer is the most common cancer diagnosed globally, with a predictable 2.3 million new cases occurring in 2020 [13]. This study deals with a relatively small sample size that limits the statistical power and generalizability of the findings, particularly regarding the estimation of future trends such as changes in mortality rates, which was out of the scope of this study due to the short period of study. The primary aim of the study was to characterize clinical patterns among patients treated at specialized oncology units, rather than to estimate population‐level prevalence. Therefore, while the results may not represent the general Iraqi population, they provide valuable insight into the clinical profile of patients who reach tertiary care settings, which is critical for understanding the burden and management needs of advanced cases in Iraq. The highest percentage of breast cancer patients (39%) belonged to the 50–59 years age group, while the lowest percentages were observed in the 21–29 years (5%) and 70–79 years categories. These findings align with previous studies suggesting that breast cancer is more prevalent in women over 50 years, correlating with postmenopausal hormonal changes that increase the risk of malignancy. At a biological level, the increased sensitivity to estrogens in epithelial breast cells, immune senescence, and modifications of the tumor microenvironment in this group of patients could play a role in the late presentation of breast cancer [14]. Another explanation could be that breast cancer screening guidelines generally do not recommend routine mammography screening in women older than 75 years and only to younger age groups [15]. The predominance of postmenopausal cases supports the established association between estrogen–progesterone imbalance and breast cancer risk. Studies have shown that extended exposure to estrogen, whether natural or drugs that contain estrogen (hormonal replacement therapy), particularly in postmenopausal women, may contribute to breast cancer development. Estrogen plays a critical role in the development, progression, and prognosis of breast cancer, mainly in hormone estrogen receptor–positive tumors. Estrogen regulates cellular growth and differentiation in breast tissue and initiates cancer development via binding to receptors and activating genes that promote cell division and stimulate signaling pathways that increase cell division, and estrogen metabolites can generate free radicals leading to DNA mutation [16]. Most cancer patients were married (78%); marriage and parity have been suggested to have a protective effect against breast cancer, likely due to pregnancy‐related hormonal changes [17]. About 29% of patients used oral contraceptives, and studies have linked prolonged use of oral contraceptives with a slight increase in breast cancer risk due to hormone exposure [18]. The prevalence of hypertension and diabetes in breast cancer is consistent with research suggesting that metabolic disorders and insulin resistance contribute to breast cancer progression by promoting chronic inflammation and cell proliferation [19]. The lack of statistical significance (NS) suggests that genetic predisposition, while an important risk factor, may not be the sole determinant in breast cancer development, emphasizing the role of lifestyle and environmental factors [20]. Most of them were from Baghdad, which could be attributed to a higher healthcare accessibility, lifestyle factors, and increased environmental exposure to risk factors in urban areas. The predominance of participants from Baghdad (93% urban) limits the generalizability of our findings to rural populations, and studies are planned to include multicenter data collection from both urban and rural areas to enhance external validity and representativeness. The findings of this study suggest that a significant proportion of cases are detected in early stages (I and II: 69%); a notable percentage (31%) is diagnosed at advanced stages (III and IV), emphasizing the need for early screening programs using ultrasound or mammography and awareness workshops and campaigns. Our study population was derived from hospital‐based records, which inherently includes a higher proportion of patients with advanced or metastatic disease due to referral patterns at tertiary centers, and these cases with metastatic breast cancer, which is a major clinical challenge, often require multimodal treatment approaches, including chemotherapy, targeted therapy, and hormonal treatments. The blood group distribution among breast cancer patients was O+ (17%) and A+ (15%) were the most common; while some studies suggest a potential link between blood group and cancer susceptibility, no definitive evidence confirms an association between specific blood groups and breast cancer risk or prognosis [21]. All patients underwent CT and mammography, reflecting the standard diagnostic approach for breast cancer detection and staging. Mammography remains the gold standard for early detection, while CT scans assist in staging and evaluating metastasis [22]. Chemotherapy (50%) is the primary systemic treatment for advanced/metastatic cases that controls disease spread and prolongs survival in advanced‐stage cancer through eliminating microscopic cancer cells and reducing the risk of recurrence [23]. The dominance of chemotherapy reflects the presence of advanced‐stage and metastatic cases, where systemic therapy is prioritized. A 21% recurrence rate was observed, while 79% of patients remained recurrence‐free. Recurrence is influenced by multiple factors, including tumor biology, treatment response, and individual patient characteristics. Higher recurrence rates are often linked to late‐stage diagnosis, aggressive tumor subtypes, and incomplete treatment adherence. The findings underscore the need for early screening programs like ultrasound‐based shear‐wave elastography (SWE), an advanced, noninvasive technique complementary to grayscale sonography and lifestyle modifications [24]. This study employed only basic inferential analyses that are appropriate for its cross‐sectional design. However, advanced statistical models are needed such as multivariate regression or survival analyses that were not feasible in this study due to the study′s design and available data. Future longitudinal studies with larger cohorts are warranted to enable the use of Cox proportional hazards regression for survival prediction and ANOVA or multivariate models for detailed subgroup comparisons. The absence of adjustment for some potential confounding variables, such as socioeconomic and educational factors, was studied in this patient′s group due to incomplete data availability in retrospective records. Future studies should include comprehensive demographic information and apply multivariate statistical models to better control for these potential confounders. Future research will integrate advanced machine learning techniques such as random forest and gradient boosting applied to large‐scale epidemiological databases such as Surveillance, Epidemiology, and End Results (SEER) program and International Agency for Research on Cancer (IARC), a part of WHO known as GLOBOCAN, to model and predict age‐specific and demographic trends. This predictive approach will extend beyond descriptive analysis and contribute to early‐risk stratification and clinical decision support.

### 4.1. Conclusions

The study was of a descriptive nature and exploratory value in reflecting current patterns among the sampled population, rather than projecting long‐term or national trends in mortality. This study highlights the demographic and clinical characteristics of breast cancer patients, emphasizing the predominance of cases in postmenopausal women and those residing in urban areas. It highlights the prevalence of advanced‐stage and metastatic breast cancer, emphasizing the urgent need for enhanced screening and early detection.

### 4.2. Limitations of the Study

The sample size and the period duration were small. The relatively small sample size (No. = 100) limits the statistical power and generalizability of the findings, particularly regarding the estimation of future trends such as changes in mortality rates. Moreover, the cross‐sectional design limits causal inference and temporal assessment of risk factors. A prospective cohort study would be more appropriate to establish causal relationships and to monitor age‐ or hormone‐related trends over time. Future investigations are planned to adopt this design for a more robust evaluation.

## Funding

No funding was received for this manuscript.

## Conflicts of Interest

The authors declare no conflicts of interest.

## Data Availability

The data that support the findings of this study are available on request from the corresponding author. The data are not publicly available due to privacy or ethical restrictions.
